# Emergence of Relaxation Oscillations in Neurons Interacting With Non-stationary Ambient GABA

**DOI:** 10.3389/fncom.2018.00019

**Published:** 2018-04-05

**Authors:** Denis A. Adamchik, Valery V. Matrosov, Victor B. Kazantsev

**Affiliations:** Lobachevsky State University, Nizhny Novgorod, Russia

**Keywords:** neural oscillation, rate model, tonic conductance, GABA, interneurons

## Abstract

Dynamics of a homogeneous neural population interacting with active extracellular medium were considered. The corresponding mathematical model was tuned specifically to describe the behavior of interneurons with tonic GABA conductance under the action of non-stationary ambient GABA. The feedback provided by the GABA mediated transmembrane current enriched the repertoire of population activity by enabling the oscillatory behavior. This behavior appeared in the form of relaxation oscillations which can be considered as a specific type of brainwaves.

## 1. Introduction

Historically, the focus of experimental and theoretical studies of brain signaling was almost exceptionally on neurons and their networks. Being the only electrically excitable cells in the nervous system, neurons are able to communicate by receiving, processing and generating electrical signals in the form of spike trains (Nicholls et al., 2001). All other structures constituting the nervous tissue such as glial cells and extracellular matrix (ECM) until very recent decades were not taken into account in the mechanisms of information processing.

Glial cells and various exctracellular structures were primarily thought to perform a number of auxiliary functions such as trophic, supportive and immune (Allen et al., 2009). The comprehension of inalienability of glia and ECM to the neuronal signaling came with the discovery of chemical synaptic transmission machinery (Krnjević, 1974) and secretory function of astrocytes (Martin, 1992). Glia turned out to be a gigantic chemical factory of the nervous system, governing neurons and using the extracellular space as an intermediary (Barres, 1991).

At present, there has been a great number of theoretical and experimental studies devoted to neuron-glia interaction (Bezzi and Volterra, 2001). One of the most prominent concepts in the field was that of the tripartite synapse (Araque et al., 1999). Glial cells, particularly astrocytes, can effectively influence and modulate the synaptic transmission. Many aspects of such modulations were discussed in a number of computational studies (Postnov et al., 2007; Gordleeva et al., 2012; Kazantsev et al., 2012; Volman et al., 2012; Lazarevich et al., 2017).

Besides several glial cell types, the extracellular space itself can be an important player in neuronal signaling. It serves as an interstitial transport system mediating cell-to-cell communications by means of numerous active chemicals (Sykovaá and Nicholson, 2008). This type of communications is called “volume” transmission and is characterized by signal diffusion in a three-dimensional fashion within the brain extracellular fluid (Agnati et al., 1995). The “volume” transmission depends crucially upon the actual geometry of the ECS (Syková, 2004) which has great relevance for pharmacokinetics and actions of neuropsychoactive drugs (Zoli et al., 1999).

One of the major neurotransmitters in the CNS is γ-Aminobutyric acid (GABA) (Webster, 2001). It mediates intercellular communications by participating in both “wiring” and “volume” transmission (Semyanov et al., 2004). The “wiring” action of GABA is through mediating the synaptic transmission by activating the postsynaptic (phasic) GABA_*A*_-receptors. The “volume” transmission is, in its turn, carried out by “overspilled” ambient GABA which regulates neuronal excitability by creating the extra transmembrane current through extrasynaptic (tonic) GABA_*A*_-receptors.

GABA was reported to maintain the fast neuronal oscillations (gamma, 20–80 Hz) in inhibitory interneuron networks (Whittington et al., 1995; Bartos et al., 2007). In the computational study (Wang and Buzsáki, 1996), GABA_*A*_ synaptic transmission was shown to provide a suitable mechanism for synchronized gamma oscillations in a sparsely connected network of fast-spiking interneurons. Incidentally, GABA was reported to enhance collective behavior in neuronal axons (Traub et al., 2003). Specifically, gamma-frequency oscillations were demonstrated to coexist with phasic high-frequency oscillations (>90 Hz) in principal cell axon populations.

Primarily, GABA was considered to be the main inhibitory neurotransmitter in the brain until it was shown experimentally to be able to perform the bi-directional regulation of neuronal spiking activity (Song et al., 2011). Based on this experimental finding, a number of mathematical models describing the action of ambient GABA on the excitability properties of interneurons were suggested (Adamchik et al., 2015; Adamchik and Kazantsev, 2017). In Adamchik et al. (2015), the behavior of a single interneuron embedded in the extracellular space with constant ambient GABA concentration was studied. It was shown that depending on the parameters of tonic current, such as tonic conductance density and GABA reversal potential, the interneuron demonstrated different behavioral modes including self-oscillations. The impact of stationary GABA at the population level was studied subsequently in Adamchik and Kazantsev (2017). Specifically, it led to bistability between asynchronous firing and zero-activity state.

In this paper, we study the effects of non-stationary, activity dependent GABA upon population dynamics of interneurons. To this end, we propose a mathematical model accounting for the feedback between interneurons and ambient GABA (section 2). The origin of the feedback has the following explanation. Extracellular GABA creates the additional transmembrane current through activation of extrasynaptic (tonic) GABA_*A*_-receptors. This current further changes the firing properties of interneurons (Adamchik et al., 2015), which immediately affects the synaptic release of GABA (Destexhe et al., 1994). Since extracellular GABA concentration depends, among others, on spillover, i.e., the diffusion of the neurotransmitter out of the synaptic cleft (Semyanov et al., 2004), it changes, which futher affects tonic conductance and provides the respective feedback.

Based on these considerations, we proposed a mathematical model using the following assumptions. First, we considered a particular case of a homogeneous population of interneurons which allowed us to describe their collective behavior using the simple rate-based formalism. Second, we neglected any spatial gradient of neurotransmitter, considering its concentration to be uniformly distributed over the entire extracellular space. This assumption allowed us to build the minimal model of the feedback avoiding dealing with an explicit model of spatiotemporal GABA dynamics. The model consisted of two coupled equations, one of which described the time-course of population activity (section 2.1) while the other–the concentration of ambient GABA (section 2.2). The model was explored both numerically and analytically (section 3). The results including the appearance of relaxation oscillations were discussed in (section 4).

## 2. Materials and methods

### 2.1. Population dynamics

Within the framework of rate-based formalism, a homogenous population of neurons is described by a single variable, e.g., the population activity, *A*. The rate of change of *A* is determined by the so called gain fuction *g*_λ_(*I*), which is unique for each cell type. The respective equation reads:

(1) $$\begin{array}{r} \tau_{m}\frac{dA}{dt}=-A+g_{\lambda }\left(I\right) \end{array}$$

where τ_*m*_ is the membrane time constant and *I* is the total input current an arbitrary neuron receives from the entire network. The latter is linearly dependent on population activity, *I* = *JA*, where the proportionality factor, *J*, is called coupling strength (Gerstner et al., 2014).

The gain *g*_λ_(*I*) is primarily a function of input current, *I*, but can also depend on a number of factors, collectively denoted here by λ. In our case, these are tonic conductance density, *G*, and GABA reversal potential, *E*, i.e., λ = (*E, G*).

The exact form of the gain function can be derived analytically only for a few simple neuron models, such as, for example, the quadratic integrate-and-fire (QIF), whose dimensionless normal form reads: $\dot{v}=v^{2}+\kappa$. Using separation of variables and integration over infinite potential bounds, one can get:

(2) $$\begin{array}{r} g_{\lambda }\left(I\right)=\frac{1}{\tau_{r}+\tau_{m}\kappa^{-1/2}} \end{array}$$

where the dimensionless parameter κ depends both on input current *I* and other factors. The absolute refractory period, τ_*r*_, is added to the period of oscillations to prevent the firing frequency from taking an arbitrarily large value. Note, that formula (Equation 2) is valid only for positive κ; when κ < 0 no oscillations occur and, as a result, *g*_λ_(*I*) = 0.

The gain function (Equation 2) describes qualitatively the responce of Class I excitability neurons. In these neurons, the transition from resting to spiking occurs via saddle-node on invariant circle bifurcation (SNIC), that allows them to fire with arbitrarily small frequency (Izhikevich, 2007). The Wang-Buzsáki interneuron (Wang and Buzsáki, 1996) belongs exactly to this type of neurons. In Adamchik and Kazantsev (2017), the original conductance-based model, modified in a way to account for the additional transmembrane tonic current, was reduced to the QIF neuron. The dimensionless parameter κ took the following form:

(3) $$\begin{array}{r} \kappa =-\frac{1+{\left(G/G_{m}\right)}^{2}}{4}+\frac{k}{G_{m}^{2}}\left[I+G\left(E-E_{m}\right)\right] \end{array}$$

where *E*_*m*_, *G*_*m*_ and *k* are constants listed in Table 1. The details of the reduction can be found in Adamchik and Kazantsev (2017).

**Table 1:** Model constants.

**Constant**	**Value**	**Description**
τ_*m*_	8.925 ms	Membrane time constant
τ_*r*_	0.627 ms	Absolute refractory period
*G*_*m*_	0.112 mS · cm^−2^	Conductance density at threshold
*E*_*m*_	−60.414 mV	Halfway between resting and
		threshold membrane potentials
*k*	0.0155 μA · cm^−2^ mV^−2^	Proportionality factor
α	5 mmol^−1^ ms^−1^	Forward rate
β	0.18 ms^−1^	Backward rate

Equation (1) along with the relations (Equations 2, 3) describes the time-course of population activity. It contains parameters such as coupling strength, *J*, and GABA reversal potential, *E*, which can take arbitrary values but remain unchanged. Tonic conductance density, *G*, is on the contrary a variable, which depends on local GABA concentration, *C*. The form of this dependence can be determined using a common kinetic formalism (Destexhe et al., 1998). According to a simplified kinetic scheme of the GABA_*A*_-receptor, which is assumed to exist in two conformations, open (O) and closed (C), one can get:

(4) $$\begin{array}{r} G=Ḡ\frac{\alpha C}{\alpha C+\beta } \end{array}$$

where α and β are forward (activation) and backward (deactivation) rates, respectively, and Ḡ is maximum conductance density. Rate values were taken from Koch and Segev (1998) and are given in Table 1 for reference.

Note, that equation (4) does not determine a momentary but rather a stable-state value of *G*. Nevertheless, we may use it because conductance relaxation time τ_*G*_ = 1/(α*C* + β) ≤ 1/β ≈ 5 ms, which is far less than the operating time of ambient GABA concentration which amounts to hundreads of milliseconds (Semyanov et al., 2004).

### 2.2. Ambient GABA dynamics

According to Semyanov et al. (2004), extracellular GABA concentration is regulated by uptake, non-synaptic release and spillover. Uptake is carried out by GABA transporters which decrease the concentration by binding and removing GABA molecules from the extracellular space. Ambient GABA can originate from various sources. It can escape from synaptic cleft (spillover) and can be released via non-vesicular mechanism by neurons and glia. Both spillover and non-synaptic release increase ambient GABA concentration but do it differently. Unlike non-synaptic release, spillover depends crucially on synaptic dynamics and, as a result, on population activity. These general considerations allowed us to write a governing equation for ambient GABA concentration:

(5) $$\begin{array}{r} \frac{dC}{dt}=-\frac{C-C_{0}}{\tau_{C}}+S\left(A\right) \end{array}$$

The first term is supposed to describe the mutual action of uptake and non-vesicular release, which counterbalance each other by maintaining an optimal background GABA level, denoted here by *C*_0_. Spillover is described, in its turn, by the second term, *S*(*A*), representing the production function of GABA and depending explicitly on population activity. The exact form of the production function was chosen in a way to describe qualitatively correctly the properties of synaptic neurotransmitter release. In the most common case, the production function reads as follows:

(6) $$\begin{array}{r} S\left(A\right)=Q\frac{A\tau_{P}}{A\tau_{P}+1} \end{array}$$

where τ_*P*_ is GABA production time constant and *Q* is the maximum production rate.

The exact form of the production function can be derived based on the following consideration. Let δ*C*_*m*_ be the amount of GABA released in response to a single spike. Then, due to exhaustion of synaptic vesicle pools, the next spike, coming τ time units after the first one, will evoke the release of a lesser amount of neurotransmitter, precisely δ*C* = δ*C*_*m*_[1 − exp(−τ/τ_*P*_)]. For a Poisson spike train, the interspike interval distribution (ISI) with the mean firing rate equal to *A* reads: *P*(τ) = *A*·exp(*Aτ*). Then, the average amount of GABA released in response to a spike from the spike train will be $\langle \delta C\rangle =\int_{0}^{\infty }\delta C\cdot P\left(\tau \right)d\tau =\delta C_{m}/\left(1+A\tau_{P}\right)$. The product *A*·〈δ*C*〉 gives the required production rate (cf. 6), where *Q* = δ*C*_*m*_/τ_*P*_.

### 2.3. Dynamical system

Two coupled ordinary differential equations (ODE) (1, 5) with relations (2–4, 6) form a 2D dynamical system. Its state variables are population activity, *A*, and ambient GABA concentration, *C*. Besides some constants (see Table 1), the equations contain a number of free parameters, which can roughly be split into two distinct groups. The first group, (*E*, Ḡ, *J*), consists of the parameters controlling population activity, while the second one, (*C*_0_, *Q*), determines ambient GABA concentration. Our task is to reveal how the dynamics of equations (1, 5) depend on all these parameters. Some preliminary considerations concerning the matter are the following.

In absense of the second equation (Equation 5), the system reduces to a simple 1D phase line, corresponding to the case of stationary external medium. This particular case was a subject of our previous study (Adamchik and Kazantsev, 2017). It was shown then that introduction of tonic current did not lead to any new dynamical effects compared to the reference case, (*G* = 0), characterized by a simple stable-state dynamics. It resulted, however, in appearance of a monostable regime of asynchronous firing once tonic current parameters, *E* and *G*, were properly tuned, specifically, in a way that the point (*E, G*) was located above a certain curve on the parameter plane. This curve was shown previously to be a border of the self-oscillatory mode in the model of a tonically driven single neuron (Adamchik et al., 2015). The impact of coupling strength, *J*, on population activity consisted, in its turn, in inducing bistability, i.e., coexistence of resting and asynchronous firing, and expanding the bistability region at the cost of trivial (zero-activity) monostable solutions.

In this study our particular interest is on the feedback-induced dynamical effects. For this purpose we specifically focus on the parameters governing ambient GABA concentration, i.e., *C*_0_ and *Q*. Note that such parameters as baseline concentation, *C*_0_, and maximum production rate, *Q*, can be controlled pharmacologically in experiments.

### 2.4. Semi-explicit model

To verify the validity of our population model prediction as well as to visualize neural oscillations we built a semi-explicit computational model of the respective spiking network. To do this we replaced the equation for the population activity (Equation 1) with an explicit spiking neural network model but preserved (Equation 5), which describes the dynamics of ambient GABA.

We considered specifically a network of *N* = 100 interneurons randomly coupled with probability *p* = 0.1. Each neuron was described by the original conductance-based model (Wang and Buzsáki, 1996) with the additional tonic current term: *I*_*GABA*_ = *G*(*u* − *E*), where *u* is the membrane potential. Tonic conductance density, *G*, depended here on extracellular GABA concentration just in accordance with equation (4). Synaptic transmission was mediated by phasic GABA_*A*_-receptors. The total synaptic current to an arbitrary neuron was determined as the normalized sum over the contributions of all its presynaptic neighbors:

(7) $$\begin{array}{r} I_{syn}=\frac{1}{M}\sum G_{syn}r\left(u-E\right) \end{array}$$

where *M* is the mean number of presynaptic inputs: *M* = *Np*. The fraction of the receptors in the open state, *r*, obeyed the kinetic equation

(8) $$\begin{array}{r} \frac{dr}{dt}=\alpha T\left(1-r\right)-\beta r \end{array}$$

while the synaptic concentration of GABA, *T*, strictly followed the potential at the presynapse:

(9) $$\begin{array}{r} T\left(u_{pre}\right)=\frac{T_{max}}{1+\exp\left(-\frac{u_{pre}-\Theta }{\Delta }\right)} \end{array}$$

Besides transmembrane and synaptic components, the total current to each neuron included also a constant one, *I*_0_, which served to regulate the level of depolarization. The parameters of synaptic transmission are listed in Table 2.

**Table 2:** Model synapse.

**Parameter**	**Value**	**Description**
*G*_*syn*_	0.1 mS · cm^−2^	Maximum phasic (synaptic) conductance density
*T*_*max*_	1 mmol	Peak synaptic cleft concentration of GABA
Θ	0 mV	Threshold of the activation
Δ	2 mV	Width of the transition area
*I*_0_	−1.4 μA cm^−2^	Depolarization current

To couple the explicit spiking network model with the equation describing ambient GABA dynamics we calculated at each time step the instantaneous population activity, using for averaging the α-function (Dayan and Abbott, 2001):

(10) $$A(t)=\frac{1}{N}\sum_{\begin{array}{c} s=j\tau_{s},\\ j\in N \end{array}}^{3\tau_{w}}\alpha (s)\;S(t-s;t-s+\tau_{s}),$$

(11) $$\alpha (s)={\left[\frac{s}{\tau_{w}^{2}}\exp\left(-\frac{s}{\tau_{w}}\right)\right]}_{+}$$

Here, *S*(*t* − *s*; *t* − *s* + τ_*s*_) is the total number of spikes, the entire network generates within the respective time window; τ_*w*_ = 20 ms and τ_*s*_ = 1 ms are averaging and sliding windows, respectively.

## 3. Results

### 3.1. Numerical and phase plane analysis

**Figure 1:**
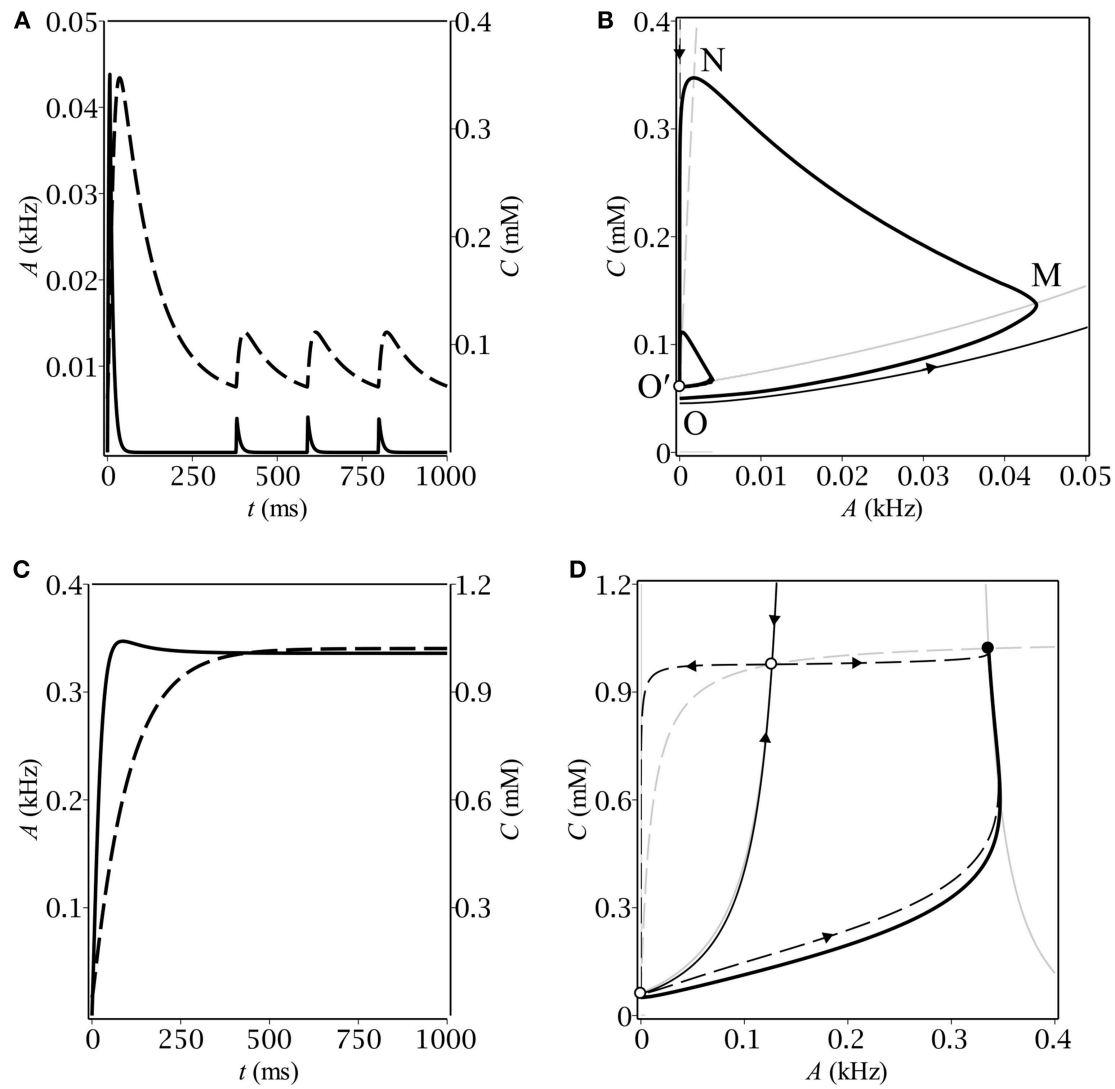
The time-course of population activity (solid) and ambient GABA concentration (dashed) along with the respective phase plane trajectories (thick solid) for oscillatory **(A,B)** and stationary **(C,D)** modes. On the phase portraits **(B,D)**, thin gray lines correspond to A- (solid) and C- (dashed) nullclines, respectively. Separatrices of the saddle are represented by a pair of thin black lines with arrows indicating the flow direction.

First, we performed numerical analysis of the dynamical system (Equations 1, 5). We carried out numerous simulation trials for a wide range of biologically relevant parameter values. In each trial, the system proceeded with the same initial conditions (*A*|_*t* = 0_ = 0, *C*|_*t* = 0_ = *C*_0_) corresponding to zero population activity and baseline GABA concentration, respectively. We found out that, depending on parameters, the system demonstrated either oscillatory or stationary behavior (see Figure 1). The parameters of the simulations are listed in Table 3.

**Table 3:** Model parameters.

**Parameter**	**Value**	**Description**
*J*	50 ms ·μA cm^−2^	Coupling strength
*E*	−50 mV	GABA reversal potential
Ḡ	1 mS · cm^−2^	Maximum tonic conductance density
τ_*C*_	100 ms	GABA relaxation time constant
τ_*P*_	100 ms	GABA production time constant
*C*_0_	0.05 mmol	Baseline ambient GABA concentration
*Q*	0.02 mmol · ms^−1^ (oscillations)	Maximum GABA production rate
	0.01 mmol · ms^−1^ (stationary)	

**Figure 2:**
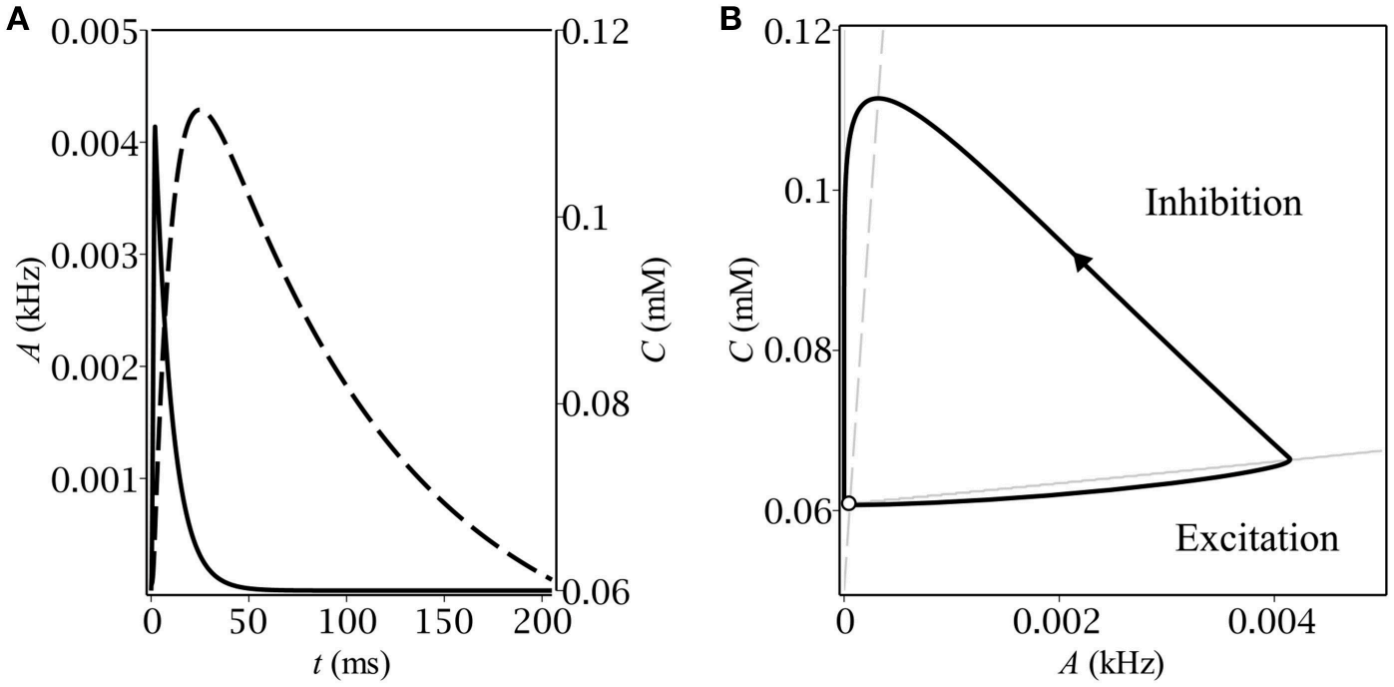
Stationary relaxation oscillations in the model of the feedback between a population of interneurons and GABA-containing extracellular medium: **(A)** the time-course of the dynamical variables during one period of oscillations–population activity (solid) and ambient GABA concentration (dashed); **(B)** the corresponding phase plane trajectory in the form of a limit cycle. The unstable fixed point (denoted by the empty circle) lies close to the limit cycle.

Different kinds of behavior can be accounted for using the phase plane. In case of oscillations, the trajectory first makes a big loop before converging to the limit cycle (see Figure 1B). The latter is intersected by the *A*-nullcline, coinciding for the small *A*'s with the border between zero and non-zero gain: *g*_λ_(0) = 0 (see Figure 2B). It means that the system in the oscillatory mode sequentially visits the region of excitatory GABA action. The oscillations would evidently not occur if the baseline GABA concentration, *C*_0_, exceeded the borderline value between inhibition and excitation, *C*_+_. In other words, if the system was placed into the region of inhibitory GABA, it would never leave it. In the stationary mode, the trajectory moves up slower than the *A*-nullcline does (see Figure 1D), so it converges to the fixed point corresponding to stationary asynchronous firing. This scenario realizes if the maximun production rate, *Q*, is lower than a certain threshold value. These considerations helped us subsequently to determine conditions for oscillations (section 3.2).

Let us now describe the biophysical mechanism underlying periodical oscillatory solutions (see Figure 1B). If the baseline ambient GABA concentration, *C*_0_, is high enough to make neurons fire but not too high to inhibit them by shunting, the initially silent neurons start firing. Non-zero population activity makes activity dependent ambient GABA concentration steadily grow up through synaptic release and spillover (OM). If parameters, governing ambient GABA dynamics, are properly tuned, then, at some point, tonic GABA switches from excitation to inhibition. On the phase plane, it corresponds to the intersection of the trajectory with the *A*-nullcline (M). As soon as the intersection occurs, the gain becomes zero and population activity starts decreasing to its steady-state (zero) value with a time constant of the membrane, τ_*m*_. While population activity goes down, the concentration keeps growing but its grow rate gradually slows down (MN). Eventually, the rate of change of *C* becomes negative and the trajectory moves down with a time constant of concentration, τ_*C*_ (NO′). At some point (O′), the trajectory re-enters the region of excitatory GABA and the entire process starts from the scratch. Note, that concentration does not reach its baseline level, *C*_0_, so the magnitude of the limit cycle is less than that of the initial loop.

To avoid the trajectory from making a loop before converging to the limit cycle, we took the initial conditions exactly at the upper border between inhibitory and excitatory GABA: *A*|_*t* = 0_ = 0, *C*|_*t* = 0_ = *C*_+_. The condition for the border follows directly from equation (1) as: *g*_*C*_+_−0_(0) > 0, *g*_*C*_+_+0_(0) = 0. Based on the explicit analytical expression for the gain function (Equations 2, 3) as well as on the relation between tonic conduction density, *G*, and GABA concentration, *C*, (Equation 4), one can get:

(12) $$\begin{array}{r} C_{\pm }=\frac{\beta }{\alpha }\frac{G_{\pm }}{Ḡ-G_{\pm }}, \end{array}$$

where

(13) $$\begin{array}{r} G_{\pm }=G_{m}\left(x\pm \sqrt{x^{2}-1}\right),\;x=\frac{2k}{G_{m}}\left(E-E_{m}\right) \end{array}$$

The minus-subscripted concentration, *C*_−_, corresponds to the transition from inhibition to excitation as we move upwards the *C*-axis and is given here just for reference. Its value for the actual choice of parameters (see Table 3) is negligible and its existence does not play any substantial role for oscillations. Oscillations occur essentially at the border between excitatory and inhibitory GABA and not vice versa.

Note, that the solution exists only if GABA reversal potential, *E*, lies above a certain threshold, whose value is determined by zero determinant condition (see equation (11)): $E^{*}=E_{m}+\frac{G_{m}}{2k}\approx -56.8$ mV, which exceeds the resting membrane potential (≈−64 mV) by 9.2 mV.

The time-course of the dynamical variables as well as the shape of the limit cycle (see Figure 2) are typical for relaxation oscillations. In the excitatory region, the population activity relaxes to the value determined by the gain function but as soon as it leaves it, *A* starts the exponential decay to zero. The concentration follows the population activity with a delay caused by the difference between the time constants of membrane, τ_*m*_, and of concentration, τ_*C*_.

### 3.2. Conditions for oscillations and their characterisctics

**Figure 3:**
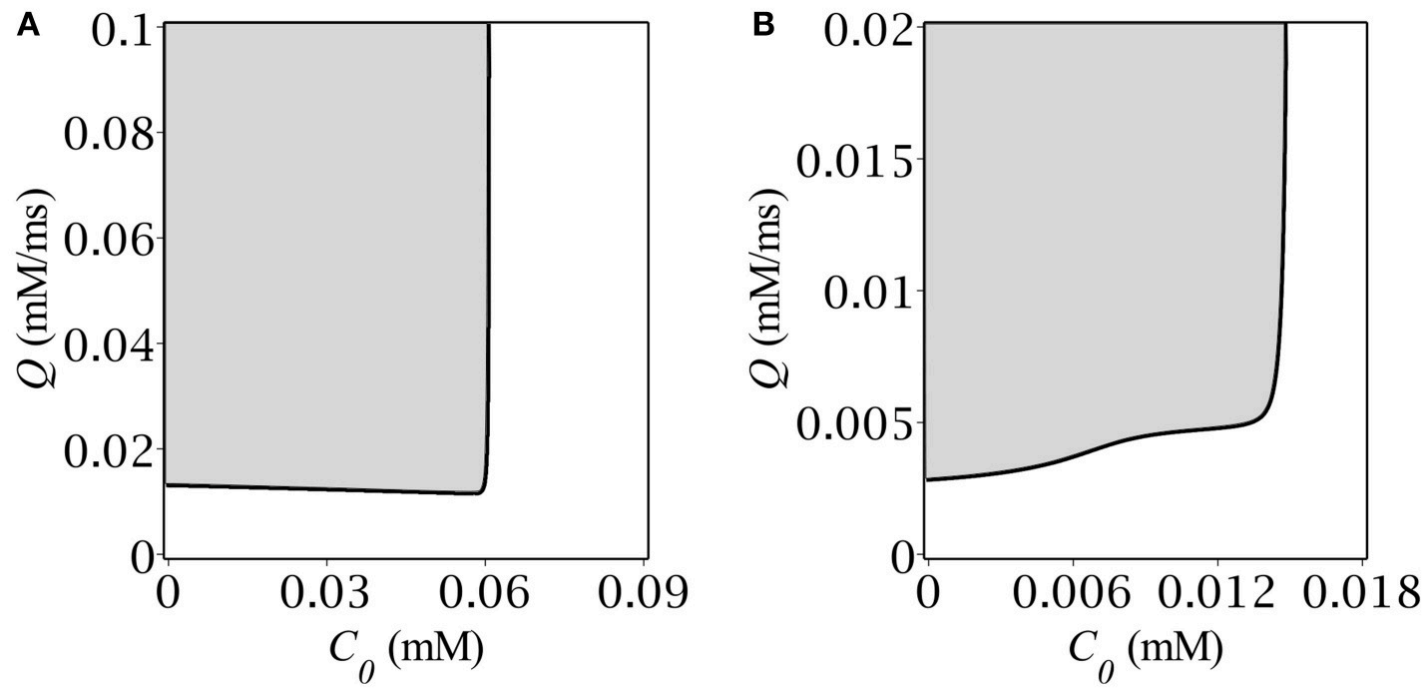
Oscillatory region on the plane (gray) (*C*_0_, *Q*) for **(A)** *E* = −50 mV, **(B)** *E* = −55 mV.

Our next step was to determine the region in the parameter plane (*C*_0_, *Q*) where the system had periodical solutions. For this purpose we implemented the following calculations. For each point (*C*_0_, *Q*) from the rectangular (0, *C*_*max*_) × (0, *Q*_*max*_) we traced the time evolution of the dynamical system Equations (1, 6) under the same initial conditions: *A*|_*t* = 0_ = 0, *C*|_*t* = 0_ = *C*_+_. Depending on the parameters, the system either remained at the starting point or relaxed to the upper stable state, or oscillated periodically. The periodicity was ascertained based on multiple crossings of the trajectory with the *A*-nullcline. The border between the regions of oscillatory and transient solutions is depicted in Figure 3 for two different values of GABA reversal potential. Specifically, Figure 3A corresponds to highly depolarizing GABA, while Figure 3B corresponds to GABA, whose reversal potential is slighly above (precisely by 1.8 mV) the oscillatory threshold.

The oscillatory region for highly depolarizing GABA has roughly the shape of a semi-infinite strip, (0, *C*_+_) × (*Q*_−_, ∞), where the border values *C*_+_ and *Q*_−_ are both dependent on the reversal potential, *E*. As far as we get closer to the oscillatory threshold, the region shrinks until collapsing at *E* = −56.8 mV. The shape of the oscillatory region for high *E*'s implies that oscillations occur if both the baseline GABA concentration and the GABA production rate are located below and above their respective threshold values: *C* < *C*_+_, *Q* > *Q*_−_. Note, that the upper bound *C*_+_ corresponds exactly to the concentration value at the starting point of simulation. The shape of the oscillatory region has the straightforward phase-plane interpretation (see section 3.1). Although, we managed to find the explicit analytical expression for *C*_+_ (see Equations 10, 11), there was no way to obtain such for *Q*_−_(*E*), the more so it depends not only on *E* but on *C*_+_ as well, which is illustrated in Figure 3B.

**Figure 4:**
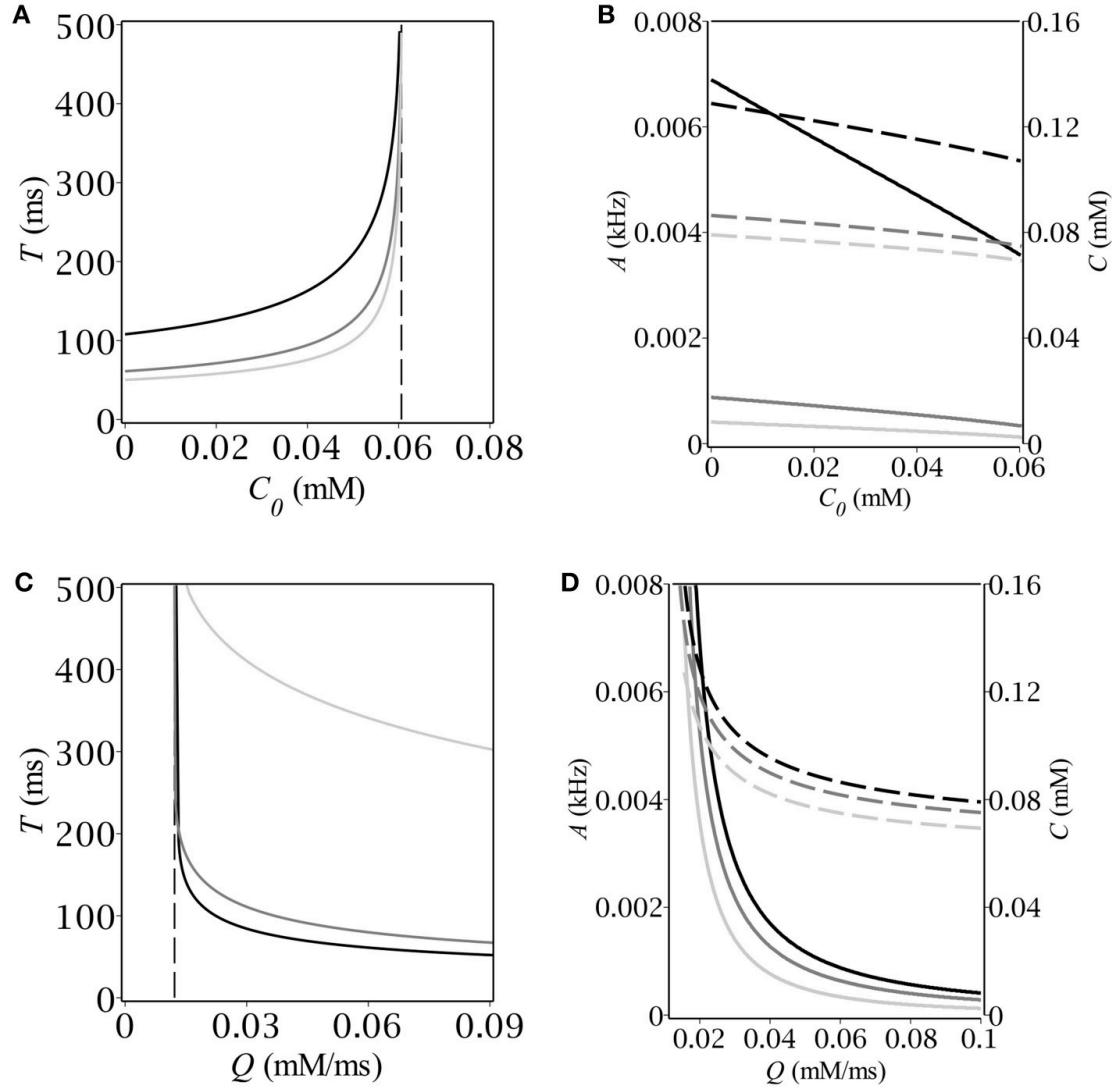
**(A,C)** Period and **(B,D)** magnitude of relaxation oscillations (A–solid, C–dash) vs. baseline GABA concentration and maximal GABA production rate. The parameters are: *E* = −50 mV, **(A,B)** *Q* [mmol · ms^−1^]: 0.02 (black), 0.06 (dim gray), 0.1 (silver); **(C,D)** *C*_0_ [mmol]: 0 (black), 0.03 (dim gray), 0.06 (silver).

Having found the oscillatory region we looked for the magnitude and period of oscillations as functions of *C*_0_ and *Q*. To this end we started time-course simulations from the same point at the limit cycle as we did before in oder to pass the transition phase. We defined the period of oscillations as the time before two subsequent intersections with the *A*-nullcline with the same sign of the slope. The magnitude of oscillations was determined as the maximum value of population activity and concentration, respectively. The results are depicted in Figure 4. First, we fixed the maximum production rate, *Q*, and found numerically the dependence of both period and magnitude on the baseline concentration of GABA, *C*_0_ (see Figures 4A,B). Next, we fixed *C*_0_ and found the respective dependencies on the parameter *Q*; the corresponding graphs are depicted in Figures 4C,D. Note, that the period grows infinitely as soon as we approach the boundaries of the oscillatory region. The dependence of the magnitude on the production rate is more drastic than that on the background GABA level. The explanation is quite simple – the higher *Q* is, the faster the periodic trajectory intersects the *A*-nullcline, the lower is the magnitude.

### 3.3. Spiking network simulation

**Figure 5:**
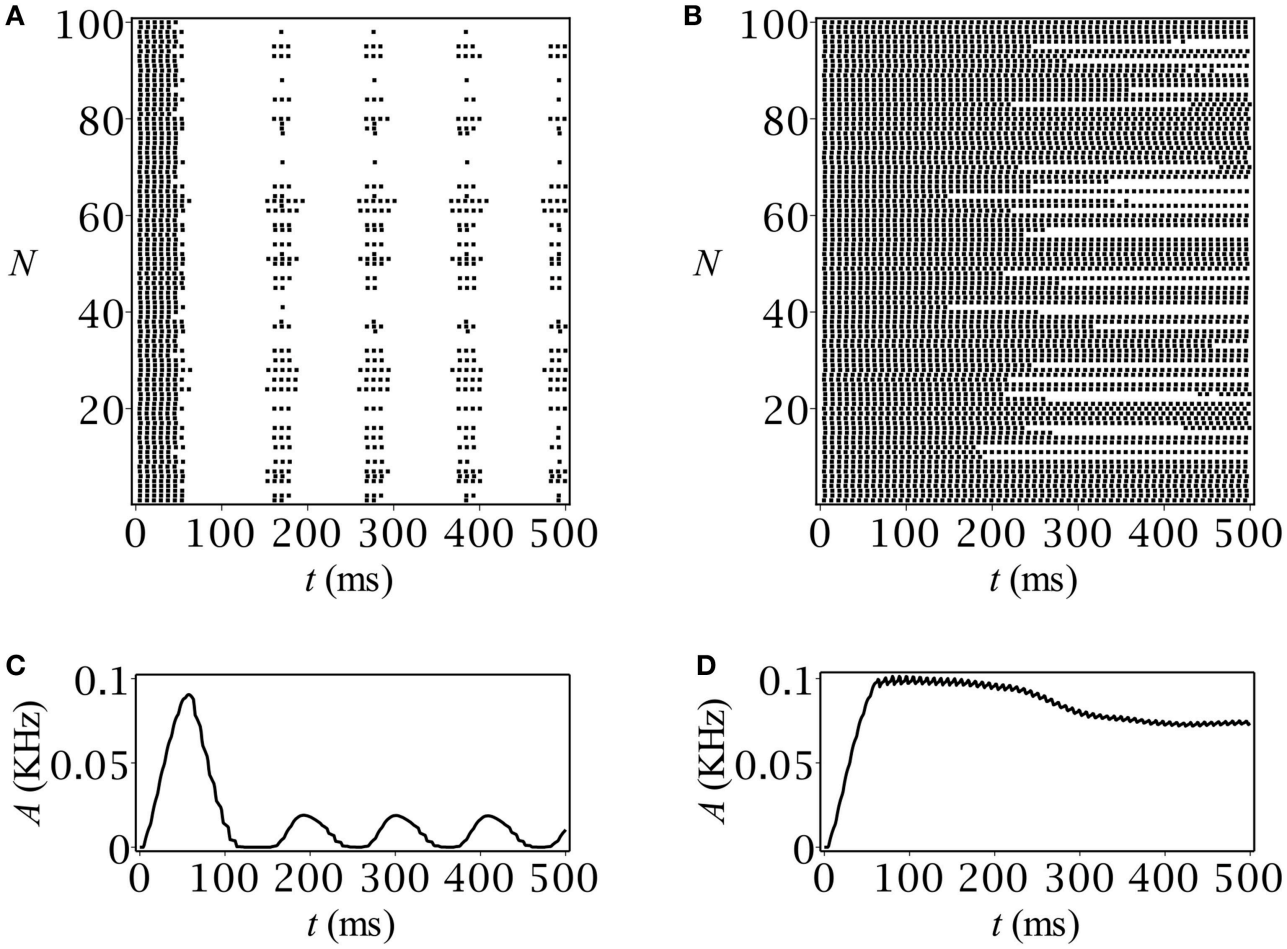
Spike raster plots and time-course of population activity for **(A,C)** oscillatory and **(B,D)** stationary modes. The parameters are: *C*_0_ = 0.05 mmol, *Q* [mM/ms]: **(A,C)** 0.01, **(B,D)** 0.003.

The results of our computer simulation are depicted in Figure 5. Note that they quite correctly reproduce those obtained using the original rate model. In case of periodicity, after an initial burst of population activity, the network demonstrates stationary oscillatory behavior (Figure 5C) just in accordance with the prediction (Figure 1A). The stationary-like behavior in the spiking network model was another option that we can verify at the network level (see Figure 5D).

## 4. Discussion

We proposed a self-consistent model of interneurons interacting with extracellular, activity dependent GABA. The model represented two coupled nonlinear ODEs describing the dynamics of population activity and ambient GABA concentration, respectively. To write the first equation we used the well-known Wilson-Cowan formalism describing the low-pass behavior of a neural ensemble (Gerstner et al., 2014). The gain function was chosen in a way to properly mimic the behavior of the interneuron with tonic GABA conductance (Adamchik and Kazantsev, 2017). The dynamics of ambient neurotransmitter were quantitatively accounted for on the basis of empirical evidence about the sources and sinks of extracellular GABA (Semyanov et al., 2004). Mathematically, the model was a continuous-time dynamical system on a plane. It was shown to admit both stationary and periodic solutions depending on the parameters governing neurotransmitter concentration. Unlike the stationary-like behavior, periodicity was a feedback-induced feature with clear biophysical explanation. In oscillatory mode, the system evolved between the regions of excitatory and inhibitory GABA. In each of these regions the dynamical variables relaxed to their stable-state values, so that the type of the oscillations was essentially relaxational. Such a pattern of synchronized population activity can be regarded as a specific type of brainwave.

We determined the conditions for oscillations and their characteristics such as period and magnitude as a function of GABA parameters. In particular, we found out that oscillations were possible only for strongly depolarizing GABA. For interneurons, GABA reversal potential had to exceed the RMP by at least 9.2 mV for oscillations to occur, which is above the reported values (Michelson and Wong, 1991; Verheugen et al., 1999; Chavas and Marty, 2003; Banke and McBain, 2006). The oscillatory region on the plane of baseline GABA concentration and maximum GABA production rate had roughly the shape of a semi-infinite stripe, i.e., there existed an upper background level of GABA and a lower intensity of its production, beyond which no oscillations occured. As a consequence, neural oscillations could be induced or suppressed pharmacologically, by changing GABA control parameters. This might have some biomedical implications since extrasynaptic GABA is believed to contribute to epileptic or schizophrenic brain activity (Brickley and Mody, 2012).

In conclusion, we need to mention the limitations of our present consideration. The suggested model of the feedback between neurons and extracellular GABA is minimal in the sense that it does not account for many key features of real neural networks and their environment. For example, when discussing ambient GABA dynamics (section 2.2) we assumed GABA sources, sinks, and receptors to be co-local. This allowed us to describe the time evolution of ambient GABA concentration with a simple ODE instead of building a detailed model accounting for the actual geometry of the extracellular space. Futher, we assumed uptake and non-vesicular release independent on population activity, although there is experimental evidence that this traditional view was too simplistic (Richerson and Wu, 2003). In addition, we considered the special case of a homogeneous neural network which is a rough representation of real neuronal ensembles. Taking into view all these considerations, we must admit that our conclusions can offer only primary insights into the feedback-induced dynamics of GABA-driven interneurons. At the same time, they can be regarded as reference point for future studies applying more sophisticated methods.

## Author contributions

DA designed the model and the computational framework, carried out the implementation and wrote the manuscript. VM contributed to the interpretation of the results. VK conceived the study and was in charge of overall direction and planning. All authors provided critical feedback and helped shape the research, analysis and manuscript.

### Conflict of interest statement

The authors declare that the research was conducted in the absence of any commercial or financial relationships that could be construed as a potential conflict of interest. The reviewer CB and handling Editor declared their shared affiliation.

## References

[B1] Adamchik D. A. Kazantsev V. B. (2017). Tonic regulation of stationary asynchronous firing of a neural network. J. Comput. Neurosci. 43, 107–114. 10.1007/s10827-017-0648-6 28509116

[B2] Adamchik D. A. Matrosov V. V. Semyanov A. V. Kazantsev V. B. (2015). Model of self-oscillations in a neuron generator under the action of an active medium. JETP Lett. 102, 624–627. 10.1134/S0021364015210031

[B3] Agnati L. F. Zoli M. Strömberg I. Fuxe K. (1995). Intercellular communica tion in the brain: wiring versus volume transmission. Neuroscience 69, 711–726. 10.1016/0306-4522(95)00308-6 8596642

[B4] Allen N. J. Barres B. A. (2009). Neuroscience: glia–more than just brain glue. Nature 457, 675–677. 10.1038/457675a 19194443

[B5] Araque A. Parpura V. Sanzgiri R. P. Haydon P. G. (1999). Tripartite synapses: glia, the unacknowledged partner. Trends Neurosci. 22, 208–215. 10.1016/S0166-2236(98)01349-6 10322493

[B6] Banke T. G. McBain C. J. (2006). GABAergic input onto CA3 hippocampal interneurons remains shunting throughout development. J. Neurosci. 26, 11720–11725. 10.1523/JNEUROSCI.2887-06.2006 17093093 PMC6674795

[B7] Barres B. A. (1991). New roles for glia. J. Neurosci. 11, 3685–3694. 1720814 10.1523/JNEUROSCI.11-12-03685.1991 PMC6575284

[B8] Bartos M. Vida I. Jonas P. (2007). Synaptic mechanisms of synchronized gamma oscillations in inhibitory interneuron networks. Nat. Rev. Neurosci. 8, 45. 10.1038/nrn2044 17180162

[B9] Bezzi P. Volterra A. (2001). A neuron-glia signalling network in the active brain. Curr. Opin. Neurobiol. 11, 387–394. 10.1016/S0959-4388(00)00223-3 11399439

[B10] Brickley S. G. Mody I. (2012). Extrasynaptic GABAA receptors: their function in the CNS and implications for disease. Neuron 73, 23–34. 10.1016/j.neuron.2011.12.012 22243744 PMC3399243

[B11] Chavas J. Marty A. (2003). Coexistence of excitatory and inhibitory GABA synapses in the cerebellar interneuron network. J. Neurosci. 23, 2019–2031. 12657660 10.1523/JNEUROSCI.23-06-02019.2003 PMC6742031

[B12] Dayan P. Abbott L. F. (2001). Theoretical Neuroscience, Vol. 806 Cambridge, MA: MIT Press.

[B13] Destexhe A. Mainen Z. F. Sejnowski T. J. (1994). Synthesis of models for excitable membranes, synaptic transmission and neuromodulation using a common kinetic formalism. J. Comput. Neurosci. 1, 195–230. 10.1007/BF00961734 8792231

[B14] Destexhe A. Mainen Z. F. Sejnowski T. J. (1998). Kinetic models of synaptic transmission. Methods Neuronal Model. 2, 1–25.

[B15] Gerstner W. Kistler W. M. Naud R. Paninski L. (2014). Neuronal Dynamics: from Single Neurons to Networks and Models of Cognition. New York, NY: Cambridge University Press.

[B16] Gordleeva S. Y. Stasenko S. V. Semyanov A. V. Dityatev A. E. Kazantsev V. B. (2012). Bi-directional astrocytic regulation of neuronal activity within a network. Front. Comput. Neurosci. 6:92. 10.3389/fncom.2012.00092 23129997 PMC3487184

[B17] Izhikevich E. M. (2007). Dynamical Systems in Neuroscience. Cambridge, MA; London: MIT press.

[B18] Kazantsev V. Gordleeva S. Stasenko S. Dityatev A. (2012). A homeostatic model of neuronal firing governed by feedback signals from the extracellular matrix. PLoS ONE 7:e41646. 10.1371/journal.pone.0041646 22848555 PMC3407243

[B19] Koch C. Segev I. (eds.). (1998). Methods in Neuronal Modeling: From Ions to Networks. Cambridge, MA: MIT press.

[B20] Krnjević K. (1974). Chemical nature of synaptic transmission in vertebrates. Physiol. Rev. 54, 418–540. 10.1152/physrev.1974.54.2.418

[B21] Lazarevich I. A. Stasenko S. V. Kazantsev V. B. (2017). Synaptic multistability and network synchronization induced by the neuron-glial interaction in the brain. JETP Lett. 105, 210–213. 10.1134/S0021364017030092

[B22] Martin D. L. (1992). Synthesis and release of neuroactive substances by glial cells. Glia 5, 81–94. 10.1002/glia.440050202 1349588

[B23] Michelson H. B. Wong R. K. (1991). Excitatory synaptic responses mediated by GABAA receptors in the hippocampus. Science 253, 1420–1423. 10.1126/science.1654594 1654594

[B24] Nicholls J. G. Martin A. R. Wallace B. G. Fuchs P. A. (2001). From Neuron to Brain, Vol. 271 Sunderland, MA: Sinauer Associates.

[B25] Postnov D. E. Ryazanova L. S. Sosnovtseva O. V. (2007). Functional modeling of neuralglial interaction. Biosystems 89, 84–91. 10.1016/j.biosystems.2006.04.012 17320272

[B26] Richerson G. B. Wu Y. (2003). Dynamic equilibrium of neurotransmitter transporters: not just for reuptake anymore. J. Neurophysiol. 90, 1363–1374. 10.1152/jn.00317.2003 12966170

[B27] Semyanov A. Walker M. C. Kullmann D. M. Silver R. A. (2004). Tonically active GABA A receptors: modulating gain and maintaining the tone. Trends Neurosci. 27, 262–269. 10.1016/j.tins.2004.03.005 15111008

[B28] Song I. Savtchenko L. Semyanov A. (2011). Tonic excitation or inhibition is set by GABAA conductance in hippocampal interneurons. Nat. Commun. 2, 376. 10.1038/ncomms1377 21730957 PMC3144593

[B29] Syková E. (2004). Extrasynaptic volume transmission and diffusion parameters of the extracellular space. Neuroscience 129, 861–876. 10.1016/j.neuroscience.2004.06.077 15561404

[B30] Sykovaá E. Nicholson C. (2008). Diffusion in brain extracellular space. Physiol. Rev. 88, 1277–1340. 10.1152/physrev.00027.2007 18923183 PMC2785730

[B31] Traub R. D. Cunningham M. O. Gloveli T. LeBeau F. E. Bibbig A. Buhl E. H.. (2003). GABA-enhanced collective behavior in neuronal axons underlies persistent gamma-frequency oscillations. Proc. Natl. Acad. Sci. U.S.A. 100, 11047–11052. 10.1073/pnas.1934854100 12960382 PMC196924

[B32] Verheugen J. A. Fricker D. Miles R. (1999). Noninvasive measurements of the membrane potential and GABAergic action in hippocampal interneurons. J. Neurosci. 19, 2546–2555. 10087068 10.1523/JNEUROSCI.19-07-02546.1999 PMC6786065

[B33] Volman V. Bazhenov M. Sejnowski T. J. (2012). Computational models of neuron-astrocyte interaction in epilepsy. Front. Comput. Neurosci. 6:58. 10.3389/fncom.2012.00058 23060780 PMC3459315

[B34] Wang X. J. Buzsáki G. (1996). Gamma oscillation by synaptic inhibition in a hippocampal interneuronal network model. J. Neurosci. 16, 6402–6413. 8815919 10.1523/JNEUROSCI.16-20-06402.1996 PMC6578902

[B35] Webster R. (ed.). (2001). Neurotransmitters, Drugs and Brain Function. Hoboken, NJ: John Wiley and Sons.

[B36] Whittington M. A. Traub R. D. Jefferys J. G. (1995). Synchronized oscillations in interneuron networks driven by metabotropic glutamate receptor activation. Nature 373, 612. 10.1038/373612a0 7854418

[B37] Zoli M. Jansson A. Syková E. Agnati L. F. Fuxe K. (1999). Volume transmission in the CNS and its relevance for neuropsychopharmacology. Trends Pharmacol. Sci. 20, 142–150. 10.1016/S0165-6147(99)01343-7 10322499

